# MHD micropolar hybrid nanofluid flow over a flat surface subject to mixed convection and thermal radiation

**DOI:** 10.1038/s41598-022-21255-8

**Published:** 2022-10-14

**Authors:** Showkat Ahmad Lone, Maryam Ahmed Alyami, Anwar Saeed, Abdullah Dawar, Poom Kumam, Wiyada Kumam

**Affiliations:** 1 Department of Basic Sciences, College of Science and Theoretical Studies, Saudi Electronic University, (Jeddah-M), Riyadh, 11673 Saudi Arabia; 2 Department of Mathematics, Faculty of Sciences, University of Jeddah, Jeddah, Saudi Arabia; 3 Center of Excellence in Theoretical and Computational Science (TaCS-CoE), Science Laboratory Building, Faculty of Science, King Mongkut’s University of Technology Thonburi (KMUTT), 126 Pracha-Uthit Road, Bang Mod, Thung Khru, Bangkok, 10140 Thailand; 4 Department of Mathematics, Abdul Wali Khan University, Mardan, 23200 Khyber Pakhtunkhwa Pakistan; 5 Department of Medical Research, China Medical University Hospital, China Medical University, Taichung, 40402 Taiwan; 6 Applied Mathematics for Science and Engineering Research Unit (AMSERU), Program in Applied Statistics, Department of Mathematics and Computer Science, Faculty of Science and Technology, Rajamangala University of Technology Thanyaburi (RMUTT), Pathum Thani, 12110 Thailand

**Keywords:** Engineering, Mathematics and computing

## Abstract

Hybrid nanofluids play a significant role in the advancement of thermal characteristics of pure fluids both at experimental and industrial levels. This work explores the mixed convective MHD micropolar hybrid nanofluid flow past a flat surface. The hybrid nanofluid flow is composed of alumina and silver nanoparticles whereas water is used as a base fluid. The plate has placed vertical in a permeable medium with suction and injection effects. Furthermore, viscous dissipation, thermal radiation and Joule heating effects are taken into consideration. Specific similarity variables have been used to convert the set of modeled equations to dimension-free form and then has solved by homotopy analysis method (HAM). It has revealed in this investigation that, fluid motion upsurge with growth in magnetic field effects and mixed convection parameter and decline with higher values of micropolar factor. Micro-rotational velocity of fluid is upsurge with higher values of micropolar factor. Thermal flow behavior is augmenting for expended values of magnetic effects, radiation factor, Eckert number and strength of heat source. The intensification in magnetic strength and mixed convection factors has declined the skin friction and has upsurge with higher values of micropolar parameter. The Nusselt number has increased with the intensification in magnetic effects, radiation factor and Eckert number.

## Introduction

The fluid flow across a fixed surface is an important topic of fluid mechanics, which was first introduced by Blasius^1^. Later on, Sakiadis^2^ modified this problem to the fluid flow over a moving surface. Such types of flow problems have received much attention of researchers, due to their tremendous applications in engineering and industries, such as plastic extrusion, continuous casting, glass fiber and crystal development^3^. The Blasius and Sakiadis idea were further explained by the number of researchers, using different type of physical impacts and techniques^4–8^. Ishak et al.^9^ documented the flow and energy propagation characteristics of an incompressible nanofluid flow across a fixed and moving plane sheets. It was perceived that the energy transport rate boosts with the inclusion of nano particulates in the base fluid. Nadeem and Hussain^10^ reported the energy transmission through non-Newtonian Williamson nanoliquid flow over a heat fixed surface.

The fluid that comprising of micro-scale elements and possessing internal micro-structure characteristics is termed as micropolar fluid. These fluids exhibit micro-rotational inertial characteristics and micro-rotational phenomena. Micropolar fluid has several uses inside the electronic circuits, textiles, plastic sheet, power generations turbine and other excessive heated parts of heavy machinery^11^. Many studies have been published with focal emphasis upon heat transfer characteristics by employing micropolar fluid. Magyari et al.^12^ have used micropolar fluid flow with thermal flow through permeable stretched surface. Modather et al.^13^ provided a mathematical solution to the issue of energy and mass transmission of an oscillating two-dimensional electrically conducting micropolar fluid across an infinitely moving porous surface. Li et al.^14^ scrutinized heat transfer for micropolar hybrid nanofluid flow over an extending sheet and have exposed that augmentation in micropolar parameter and concentration of nanoparticles have upsurge the micropolar function. Bilal et al.^15^ discussed the influence of activation energy overheat transfer for micropolar fluid by using different properties and have highlighted that fluid flow speed has weakened with expansion in viscosity parameter, whereas thermal profiles have retarded with higher values of thermal conductivity factor. Krishna et al.^16^ have observed micropolar fluid flow with Darcy–Forchheimer model amid two rotary plates and have deduced that motion of fluid has weakened with expanding values of inertial factor.

The suspension of a single kind of small sized particle in a pure fluid for improvement of its thermal conductance is characterized as nanofluid. It has demonstrated experimentally that; such fluids have higher thermal conductivity. The small sized particles are known as nanoparticles. The idea of suspending nanoparticles into pure fluid was floated first by Choi and Eastman^17^ for improvement of the thermal conductivity of base fluid. An innovative category of fluid called hybrid nanofluid is helpful for the transition of energy. Numerous thermal applications, including freezing, renewable power, hvac systems, warm air converters, air conditioning units, transceivers, motorized industry, rechargeable cooler, ionizing radiation systems, vessels, and bioengineering, can make use of hybrid nanoliquids. Bhatti et al.^18^ have inspected the MHD nano-liquid flow amid two rotary surfaces with influence of microorganism inside the plates and revealed that fluid flow has declined, and thermal characteristics have enhanced with growth in volumetric fraction. Shah et al.^19^ forwarded the idea of Srinivas et al.^20^ by taking the impact of micropolar nanofluid flow with gold nanoparticles inside two plates and revealed that higher values of magnetic parameter has retarded the linear motion of fluid but has upsurge the micro-rotational motion. With the passage of time, it has noticed by the researchers and scientists that hanging of two distinct kinds of nanoparticles into pure fluid has augmented the thermal conductivity of pure fluids to better level. It has further established in various investigations^21–27^, that thermal flow performance of hybrid nanofluid is much better than traditional nanofluids. Bilal et al.^28^ analyzed the influence of variations in thermal conductance and heat generation for MHD hybrid nanomaterial through a permeable medium by employing slip condition and have concluded that with upsurge in nanoparticles concentration the Nusselt number has enlarged for shrinking case and has declined for stretching case. Salahiddin et al.^29^ have examined the features of hybrid nanofluid in the closed vicinity of a highly magnetized cylinder. Alharbi et al.^30^ has investigated numerically the effects of hybrid nanofluid upon an extending plate.

In the Joule heating phenomenon, electric energy is transformed to thermal energy because of resistive force to flow of current. The idea was first discovered by Joule^31^ in 1840. For its important applications in heat transfer phenomena, many investigations have been conducted keeping in mind the Joule heating effects. Loganathan and Rajan^32^ have inspected the irreversibility production for Williamson nanofluid flow subject to Joule heating influences with a conclusion that the thermal and mass flow rates is in inverse proportion of Weissenberg number. Nayak et al.^33^ has analyzed fluid flow and heat transmission impact in a microchannel of hyperbolic form, packed with power law fluid subject to influence of Joule heating and have revealed that thermal flow rate of classical non-Newtonian and Newtonian fluids has augmented with upsurge in Joule heating factor and reduction in power index. Zhou et al.^34^ examined the entropy production for MHD Newtonian fluid flow with influence of Joule heating and Darcy–Forchheimer model for flow system. Hafeez et al.^35^ have discussed the influence of Joule heating upon Cattaneo–Christov double diffusion non-Newtonian fluid flow and have concluded that augmentation in solutal relaxation time and thermal factors have declined the profiles of concentration and temperature. Shamshuddin and Eid^36^ have inspected effects of Joule heating upon mixed convective nanofluid flow over a stretched sheet and highlighted that Nusselt number has enlarged while fluid’s velocity has weakened with growth in volumetric fraction.

Thermal radiation is one of several modes of thermal transmission and plays a substantial part in heat transfer and fluid flow problems. This term has achieved a remarkable progress in the field of thermal engineering and thermal sciences. Pramanik^37^ has inspected thermal flow of fluid past an exponentially porous sheet using the influence of thermal radiations and has revealed that rate of thermal flow has enhanced for growth in thermal radiation and Casson factor. Khan et al.^38^ have deliberated thermal and mass transmission for bioconvection nanofluid flow on Riga plate with influences of nonlinear thermal radiations. The authors of this work have established that concentration of fluid has enlarged with growth in thermophoretic and activation energy parameters whereas thermal flow profiles have been upsurge with various flow factors such as growth in thermophoresis, Brownian motion, magnetic and thermal radiation parameters. Ahmad et al.^39^ have simulated the thermal radiations influence upon micropolar fluid flow in a permeable channel. Ibrahim et al.^40^ have analyzed numerically two-dimensional unsteady fluid flow with thermally radiated effects upon flow system and concluded that temperature profiles have amplified with augmentation in radiation factor and Ecker number. Shaw et al.^41^ investigated MHD hybrid nanofluid flow subject to quadratic as well as nonlinear thermal radiations. Similar studies can be seen in Refs.^42–46^.

Viscous dissipation performs as an energy source, modifying the temperature distribution and hence the heat transfer rate. Viscous dissipation is generated during deformation of fluid and connects the thermodynamics with dynamics. Waini et al.^47^ has discussed micropolar hybrid nanofluid flow upon a stretching surface subject to viscous dissipation and thermophoresis effects and have explored that with upsurge in viscous dissipation parameter the thermal flow profiles have declined. Muntazir et al.^48^ have scrutinized the MHD flow of fluid past a porous extending surface with impact of viscous dissipation and thermal radiations and have concluded that velocity, thermal as well as concentration characteristics have weakened with expansion in the values of time dependent and suction/injection parameters. Algehyne et al.^49^ have deliberated the influence of viscous dissipation and nonlinear thermal radiations upon micropolar MHD fluid flow and have established that fluid flow has decayed with growth in magnetic parameter while thermal characteristics have enlarged with upsurge in electric, magnetic, thermal ratio and radiation factors. Upreti et al.^50^ have evaluated the influence of suction and viscous dissipation for Sisko fluid flow upon stretched sheet and have highlighted that in the occurrence of thermal source the thermal flow rate has enhanced with growth in Biot number. Yaseen et al.^51^ have deliberated hybrid nanofluid flow upon a thick concave/convex shaped subject to influences of Ohmic heating and viscous dissipation. Elattar et al.^52^ have analyzed numerically the thermal flow related to the influence of viscous dissipation and Joule heating. The readers can further study the similar concept in Refs.^53–56^.

Being inspired by the above literature survey, the authors have verified that there is little work based on the micropolar hybrid nanofluid flow containing silver and alumina nanoparticles. The authors have considered the micropolar hybrid nanofluid flow containing silver and alumina nanoparticles past a stagnation point of a flat surface. The surface is placed vertical in a permeable medium with suction and injection effects. The mixed convection phenomenon is also taken into consideration. Furthermore, the viscous dissipation, thermal radiation and Joule heating impacts are taken into consideration. The present investigation is composed of problem formulation, which is shown in section “Problem formulation”, method of solution is presented in section “HAM solution”, results and discussion is presented in section “Results and discussion”, and the concluding remarks are displayed in section “Conclusion”.

## Problem formulation

**Figure 1:**
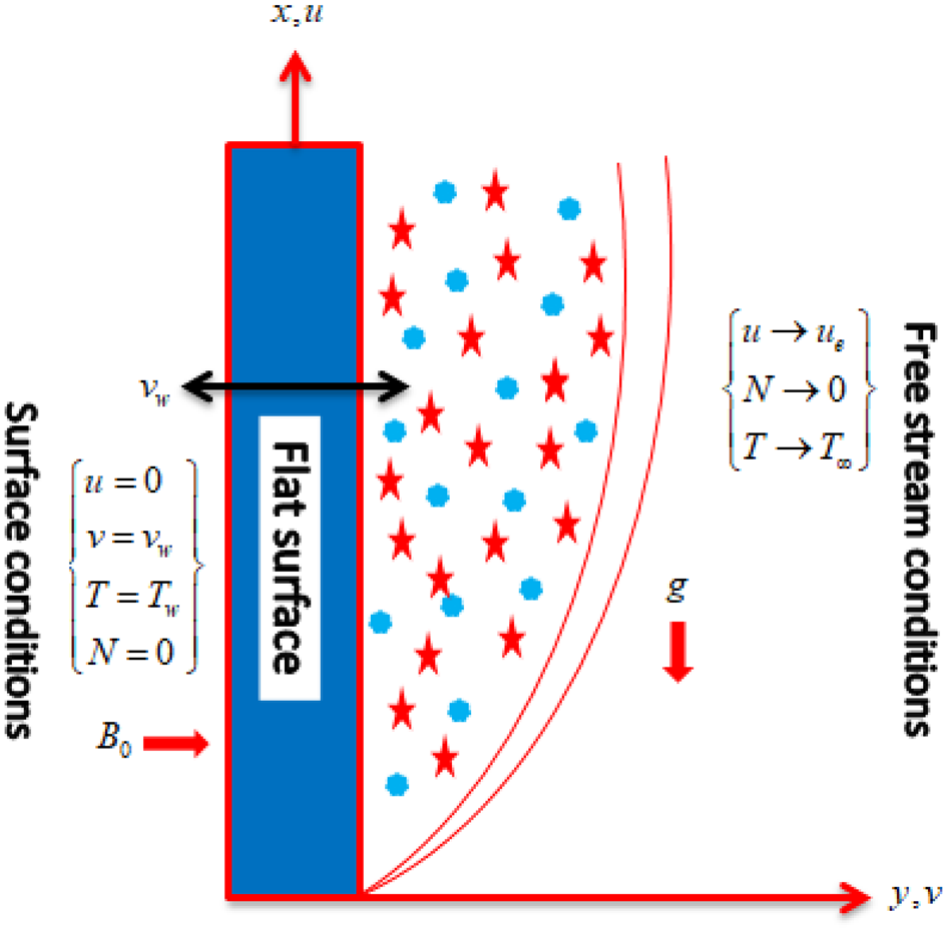
Flow geometry.

Consider the mixed convective MHD flow of a micropolar hybrid nanofluid at a stagnation point of flat sheet. The alumina and silver nanoparticles are mixed with water. $B_{0}$ is taken as strength of magnetic effects in the normal direction to fluid flow as shown in Fig. 1. The ambient motion is $u_{e} (x) = cx$, where $c$ as positive constants. At the surface of sheet, the temperature is taken as $T_{w} (x) = T_{\infty } + bx$, where $T_{\infty }$ and $b$ are the ambient temperature and concentration, respectively. Further assumptions are considered as:

- Micropolar fluid is considered at a stagnation point.
- Mixed convection effects with heat source are employed to the flow system.
- Suction/injection, viscous dissipation, thermal radiations and Joule heating effects are taken into consideration.

Equations that administered flow problem are given as^57,58^:

1 $$ \frac{\partial u}{{\partial x}} + \frac{\partial v}{{\partial y}} = 0, $$

2 $$ u\,\,\frac{\partial u}{{\partial x}} + v\,\,\frac{\partial \,u}{{\partial y}} = u_{e} \frac{{du_{e} }}{dx} + \frac{1}{{\rho_{hnf} }}\left( {\mu_{hnf} + K_{1} } \right)\frac{{\partial^{2} u}}{{\partial y^{2} }} + \frac{{K_{1} }}{{\rho_{hnf} }}\frac{\partial N}{{\partial y}} - \frac{{\sigma_{hnf} }}{{\rho_{hnf} }}B_{0}^{2} \left( {u - u_{e} } \right) + \frac{{g\left( {\rho \beta } \right)_{hnf} }}{{\rho_{hnf} }}\left( {T - T_{\infty } } \right), $$

3 $$ u\frac{\partial N}{{\partial x}} + v\frac{\partial N}{{\partial y}} = \frac{{\gamma_{hnf} }}{{\rho_{hnf} }}\frac{{\partial^{2} N}}{{\partial y^{2} }} - \frac{{K_{1} }}{{\rho_{hnf} j}}\left( {2N + \frac{\partial u}{{\partial y}}} \right), $$

4 $$ u\frac{\partial T}{{\partial x}} + v\frac{\partial T}{{\partial y}} = \frac{{k_{hnf} }}{{\left( {\rho C_{p} } \right)_{hnf} }}\frac{{\partial^{2} T}}{{\partial y^{2} }} - \frac{1}{{\left( {\rho C_{p} } \right)_{hnf} }}\frac{{\partial q_{r} }}{\partial y} + \frac{{Q_{0} }}{{\left( {\rho C_{p} } \right)_{hnf} }}\left( {T - T_{\infty } } \right) + \frac{{\mu_{hnf} }}{{\left( {\rho C_{p} } \right)_{hnf} }}\left( {\frac{\partial u}{{\partial y}}} \right)^{2} + \frac{{\sigma_{hnf} }}{{\left( {\rho C_{p} } \right)_{hnf} }}B_{0}^{2} u^{2} , $$

Subject to the following boundary conditions:

5 $$ \left\{ {\begin{array}{*{20}c} {u = 0,\,\,\,v = v_{w} ,\,\,T = T_{w} ,\,\,\,N = 0} & {\quad {\text{at}}\,\,\, \, y = 0,} \\ {u \to u_{e} ,\,\,\,\,\,\,N \to 0,\,\,\,\,\,\,T \to T_{\infty } } & {\quad {\text{as }}\,\,\,\,y \to \infty .} \\ \end{array} } \right\} $$

In Eq. (4), $q_{r}$ is expressed mathematically as:

6 $$ q_{r} = - \left( {\frac{{4\sigma^{*} }}{{3k^{*} }}\,\,\frac{{\partial \,\,T^{4} }}{\partial \,\,y}} \right). $$

By using Taylor expansion, $T^{4}$ is reduced as,

7 $$ T^{4} \,\,\, \cong \,\,\,4TT_{\infty }^{3} - 3T_{\infty }^{4} . $$

Thus, Eq. (4) is reduced as:

8 $$ u\frac{\partial T}{{\partial x}} + v\frac{\partial T}{{\partial y}} = \left( {\frac{{k_{hnf} }}{{\left( {\rho C_{p} } \right)_{hnf} }} + \frac{1}{{\left( {\rho C_{p} } \right)_{hnf} }}\frac{{16\sigma^{*} T_{\infty }^{*} }}{{3k^{*} }}} \right)\frac{{\partial^{2} T}}{{\partial y^{2} }} + \frac{{Q_{0} }}{{\left( {\rho C_{p} } \right)_{hnf} }}\left( {T - T_{\infty } } \right) + \frac{{\mu_{hnf} }}{{\left( {\rho C_{p} } \right)_{hnf} }}\left( {\frac{\partial u}{{\partial y}}} \right)^{2} + \frac{{\sigma_{hnf} }}{{\left( {\rho C_{p} } \right)_{hnf} }}B_{0}^{2} u^{2} , $$

Thermophysical properties of nanofluid and hybrid nanofluid are expressed as follows with numerical values as given in Table 1.

9 $$ \left\{ \begin{gathered} \frac{{\rho_{nf} }}{{\rho_{f} }} = \left( {1 - \vartheta_{p1} } \right) + \frac{{\rho_{p1} \vartheta_{p1} }}{{\rho_{f} }},\,\,\,\,\frac{{\left( {\rho C_{p} } \right)_{nf} }}{{\left( {\rho C_{p} } \right)_{f} }} = \,\left( {1 - \vartheta_{p1} } \right) + \vartheta_{p1} \frac{{\left( {\rho C_{p} } \right)_{p1} }}{{\left( {\rho C_{p} } \right)_{f} }},\,\,\,\,\,\frac{{\left( {\rho \beta } \right)_{nf} }}{{\left( {\rho \beta } \right)_{f} }} = \left( {1 - \vartheta_{p1} } \right) + \vartheta_{p1} \frac{{\left( {\rho \beta } \right)_{p1} }}{{\left( {\rho \beta } \right)_{f} }}, \hfill \\ \frac{{\mu_{nf} }}{{\mu_{f} }} = \frac{1}{{\left( {1 - \vartheta_{p1} } \right)^{2.5} }},\,\,\,\,\frac{{\sigma_{nf} }}{{\sigma_{f} }} = 1 + \frac{{3\left[ {\left( {\frac{{\sigma_{p1} }}{{\sigma_{f} }} - 1} \right)\vartheta_{p1} } \right]}}{{\left( {\frac{{\sigma_{p1} }}{{\sigma_{f} }} + 2} \right) - \left( {\frac{{\sigma_{p1} }}{{\sigma_{f} }} - 1} \right)\vartheta_{p1} }},\,\,\,\,\frac{{k_{nf} }}{{k_{f} }} = 1 + \frac{{3\left[ {\left( {\frac{{k_{p1} }}{{k_{f} }} - 1} \right)\vartheta_{p1} } \right]}}{{\left( {\frac{{k_{p1} }}{{k_{f} }} + 2} \right) - \left( {\frac{{k_{p1} }}{{k_{f} }} - 1} \right)\vartheta_{p1} }}. \hfill \\ \end{gathered} \right\} $$

10 $$ \left\{ \begin{gathered} \frac{{k_{hnf} }}{{k_{f} }} = \frac{{\frac{{k_{p1} \vartheta_{p1} + k_{p2} \vartheta_{p2} }}{{\vartheta_{p1} + \vartheta_{p2} }} + 2k_{f} + 2\left( {k_{p1} \vartheta_{p1} + k_{p2} \vartheta_{p2} } \right) - 2\left( {\vartheta_{p1} + \vartheta_{p2} } \right)k_{f} }}{{\frac{{k_{p1} \vartheta_{p1} + k_{p2} \vartheta_{p2} }}{{\vartheta_{p1} + \vartheta_{p2} }} + 2k_{f} - 2\left( {k_{p1} \vartheta_{p1} + k_{p2} \vartheta_{p2} } \right) + \left( {\vartheta_{p1} + \vartheta_{p2} } \right)k_{f} }}, \hfill \\ \frac{{\mu_{hnf} }}{{\mu_{f} }} = \frac{1}{{\left( {1 - \vartheta_{p1} - \vartheta_{p2} } \right)^{2.5} }},\,\,\,\frac{{\rho_{hnf} }}{{\rho_{f} }} = \left( {1 - \vartheta_{p2} } \right)\left[ {\left( {1 - \vartheta_{p2} } \right) + \vartheta_{p1} \frac{{\rho_{p1} }}{{\rho_{f} }}} \right] + \vartheta_{p2} \frac{{\rho_{p2} }}{{\rho_{f} }}, \hfill \\ \frac{{\left( {\rho \beta } \right)_{hnf} }}{{\left( {\rho \beta } \right)_{f} }} = \left( {1 - \vartheta_{p2} } \right)\left[ {\left( {1 - \vartheta_{p2} } \right) + \vartheta_{p1} \frac{{\left( {\rho \beta } \right)_{p1} }}{{\left( {\rho \beta } \right)_{f} }}} \right] + \vartheta_{p2} \frac{{\left( {\rho \beta } \right)_{p2} }}{{\left( {\rho \beta } \right)_{f} }},\, \hfill \\ \frac{{\left( {\rho C_{p} } \right)_{hnf} }}{{\left( {\rho C_{p} } \right)_{f} }} = \left( {1 - \vartheta_{p2} } \right)\left[ {\left( {1 - \vartheta_{p1} } \right) + \vartheta_{p1} \frac{{\left( {\rho C_{p} } \right)_{p1} }}{{\left( {\rho C_{p} } \right)_{f} }}} \right] + \vartheta_{p2} \frac{{\left( {\rho C_{p} } \right)_{p2} }}{{\left( {\rho C_{p} } \right)_{f} }}, \hfill \\ \frac{{\sigma_{hnf} }}{{\sigma_{f} }} = 1 + \frac{{3\left( {\frac{{\sigma_{p1} \vartheta_{p1} + \sigma_{p2} \vartheta_{p2} }}{{\sigma_{f} }}} \right) - 3\left( {\vartheta_{p1} + \vartheta_{p2} } \right)}}{{2 + \left\{ {\frac{{\sigma_{p1} \vartheta_{p1} + \sigma_{p2} \vartheta_{p2} }}{{\left( {\vartheta_{p1} + \vartheta_{p2} } \right)\sigma_{f} }}} \right\} - \left\{ {\frac{{\sigma_{p1} \vartheta_{p1} + \sigma_{p2} \vartheta_{p2} }}{{\overset{\lower0.5em\hbox{$\smash{\scriptscriptstyle\frown}$}}{\sigma }_{f} }} - \left( {\vartheta_{p1} + \vartheta_{p2} } \right)} \right\}}}, \hfill \\ \end{gathered} \right\} $$

**Table 1:** Numerical values of thermophysical properties for water and nanoparticles^59^.

Material	Water	Ag	Al_2_O_3_
$\rho$	997.1	10,500	3970
$C_{p}$	4179	235	765
$k$	0.613	429	40
$\beta$	21 × 10^–5^	1.89 × 10^–5^	8.5 × 10^–6^
$\sigma$	0.05	6.30 × 10^7^	1 × 10^–10^
$\Pr$	6.2	–	–

The similarity transformations are defined as:

11 $$ u = cxf^{\prime},\,\,\,\,v = - \sqrt {c\nu_{f} } f,\,\,\,\,\xi = \sqrt {\frac{c}{{\nu_{f} }}} y,\,\,\,\,N = cx\sqrt {\frac{c}{{\nu_{f} }}} g,\,\,\,\,\theta = \frac{{T - T_{\infty } }}{{T_{w} - T_{\infty } }}. $$

Using Eq. (11), the leading equations are transformed as:

12 $$ \left( {\frac{{1 + \chi_{1} K}}{{\chi_{1} }}} \right)f^{\prime\prime\prime} + \chi_{2} \left( {1 + ff^{\prime} - f^{{\prime}{2}} } \right) + Kg^{\prime} - M\chi_{3} \left( {f^{\prime} - 1} \right) + \lambda \chi_{4} \theta = 0, $$

13 $$ \left( {\frac{{2 + \chi_{1} K}}{{\chi_{1} }}} \right)g^{\prime\prime} + \chi_{2} \left( {g^{\prime}f - gf^{\prime}} \right) - K\left( {f^{\prime\prime} + 2g} \right) = 0, $$

14 $$ \left( {\chi_{6} + Rd} \right)\theta^{\prime\prime} + \frac{Ec\Pr }{{\chi_{1} }}f^{{\prime\prime}{2}} + \chi_{3} Ec\Pr Mf^{\prime\prime} + \Pr \chi \theta + \Pr \chi_{5} \left( {f\theta^{\prime} - \theta f^{\prime\prime}} \right) = 0. $$

Subjected conditions are given as:

15 $$ \left\{ \begin{gathered} f^{\prime}\left( \xi \right) = 0,\,\,\,\,f\left( \xi \right) = S,\,\,\,\,g\left( \xi \right) = 0,\,\,\,\,\theta \left( \xi \right) = 1\,\,{\text{ at }}\,\,\,\xi { = 0,} \hfill \\ G\left( \xi \right) \to 0,\,\,\,\,f^{\prime}\left( \xi \right) \to 1,\,\,\,\,\theta \left( \xi \right) \to 0 \, \,{\text{as}}\,\,\, \, \xi \to \infty . \hfill \\ \end{gathered} \right\} $$

In the above equations, $K = K_{1} /\mu_{f}$ is the micropolar parameter, $\lambda = Gr_{x} /{\text{Re}}_{x}^{2}$ is the mixed convection parameter,$Gr_{x} = g\beta_{f}  ( {T_{w} - T_{\infty } } )x^{3} /\nu_{f}^{2}$ is the Grashof number, ${\text{Re}}_{x} = \frac{{u_{e} ( x )x}}{{\nu_{f} }}$ is the Reynolds number, $Ec = u_{e}^{2} ( x )/( {C_{p} })_{f} ( {T_{w} - T_{\infty } } )$ is the Eckert number, $Rd = 16\sigma^{*} T_{\infty }^{3} /3k_{f} k^{*}$ is the thermal radiation factor, $\chi = Q_{0} /c( {\rho C_{p} })_{f}$ is the heat source/sink factor, $S = - v_{w} /\sqrt {c\nu_{f} }$ is the suction/injection parameter, $\Pr = ( {\mu C_{p} })_{f} /k_{f}$ is the Prandtl number and $M = \sigma_{f} B_{0}^{2} /\rho_{f} c$ is the magnetic factor. It is also to be noticed that $\chi_{1} = \mu_{hnf} /\mu_{f}$, $\chi_{2} = \rho_{hnf} /\rho_{f}$, $\chi_{3} = \sigma_{hnf} /\sigma_{f}$, $\chi_{4} = ( {\rho \beta } )_{hnf} /( {\rho \beta } )_{f}$, $\chi_{5} = ( {\rho C_{p} } )_{hnf} /( {\rho C_{p} } )_{f}$ and $\chi_{6} = k_{hnf} /k_{f}$.

### Important quantities

The important quantities skin friction coefficient ($C_{fx}$) and local Nusselt number ($Nu_{x}$) are mathematically described as:

16 $$ C_{fx} = \frac{1}{{\rho_{hnf} u_{e}^{2} \left( x \right)}}\left[ {\left( {\mu_{hnf} + K_{1} } \right)\frac{\partial u}{{\partial y}} + K_{1} N} \right]_{y = 0} . $$

17 $$ Nu_{x} = - \left[ {\frac{x}{{k_{f} \left( {T_{w} - T_{\infty } } \right)}}\left( {k_{hnf} \frac{\partial T}{{\partial y}} + \frac{{16\sigma^{*} }}{{3k^{*} }}\frac{{\partial T^{4} }}{\partial y}} \right)} \right]_{y = 0} . $$

Using Eq. (11), the above equations are reduced as:

18 $$ C_{f} = \frac{1}{{\chi_{2} }}\left[ {\frac{{1 + \chi_{1} K}}{{\chi_{1} }}} \right]f^{\prime\prime}\left( 0 \right). $$

19 $$ Nu = - \left[ {\chi_{6} + Rd} \right]\theta^{\prime}\left( 0 \right). $$

where $$C_{f} = C_{fx} \sqrt {{\text{Re}}_{x} }$$ and $$Nu = \frac{{Nu_{x} }}{{\sqrt {{\text{Re}}_{x} } }}$$.

## HAM solution

In the current investigation the modeled equations have been converted to dimension-free format. The resultant equations gave rise to highly nonlinear differential equations. The HAM^60^ method has been used to solve that set resultant equations. This technique is used to evaluate the highly nonlinear differential equations of fluid flow system and other similar problems. It is a fast convergent technique and is free from selection of embedded parameter. This technique requires initial guesses and linear operators which are defined as:

20 $$ \left\{ \begin{gathered} f_{0} \left( \xi \right) = \xi - 1 + e^{ - \xi } , \hfill \\ g_{0} \left( \xi \right) = 0, \hfill \\ \theta_{0} \left( \xi \right) = e^{ - \xi } , \hfill \\ \end{gathered} \right\} $$

21 $$ \left\{ \begin{gathered} L_{f} \left( \xi \right) = \frac{{\partial^{3} f}}{{\partial \xi^{3} }} + \frac{\partial f}{{\partial \xi }}, \hfill \\ L_{g} \left( \xi \right) = \frac{{\partial^{2} g}}{{\partial \xi^{2} }} + g, \hfill \\ L_{\theta } \left( \xi \right) = \frac{{\partial^{2} \theta }}{{\partial \xi^{2} }} + \theta , \hfill \\ \end{gathered} \right\} $$

with properties:

22 $$ \left\{ \begin{gathered} L_{f} \left( {\varepsilon_{1} + \varepsilon_{2} e^{ - \xi } + \varepsilon_{3} e^{\xi } } \right) = 0, \hfill \\ g_{0} \left( {\varepsilon_{4} e^{ - \xi } + \varepsilon_{5} e^{\xi } } \right) = 0, \hfill \\ \theta_{0} \left( {\varepsilon_{6} e^{ - \xi } + \varepsilon_{7} e^{\xi } } \right) = 0, \hfill \\ \end{gathered} \right\} $$

where $\chi_{1} - \chi_{7}$ are the constant of general solution.

### Convergence analysis

**Figure 2:**
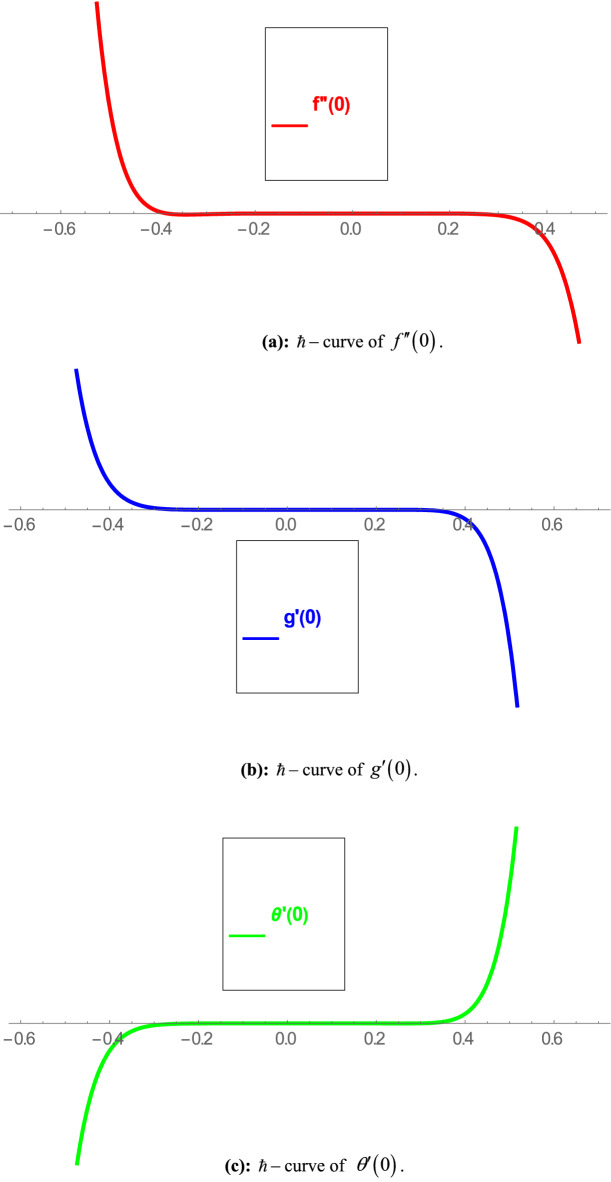
(**a**) $\hbar$-curve of $f^{\prime\prime} ( 0 )$. (**b**) $\hbar$-curve of $g^{\prime} ( 0 )$. (**c**) $\hbar$-curve of $\theta^{\prime} ( 0 )$.

In order to employ HAM, we need to determine the solutions for velocity, micro-rotational velocity and temperature in the form of series. For convergence of these solutions the auxiliary functions $\hbar_{f} ,\,\,\hbar_{g} ,\,\,\hbar_{\theta }$ come across which play a pivotal role in the convergence of method. For the purpose of this convergence, we have evaluated $\hbar$-curves at 12th order of approximations as depicted in Fig. 2a–c. Figure 2a shows the convergence of $f^{\prime\prime}( 0)$. From here, we see that $f^{\prime\prime} ( 0 )$ converges at $- 0.4 \le \hbar_{f} \le 0.3$. Figure 2b shows the convergence of $g^{\prime} ( 0 )$. From here, we see that $g^{\prime} ( 0 )$ converges at $- 0.3 \le \hbar_{g} \le 0.35$. Figure 2c shows the convergence of $\theta^{\prime} ( 0 )$. From here, we see that $\theta^{\prime} ( 0 )$ converges at $- \,0.3 \le \hbar_{\theta } \le 0.35$.

## Results and discussion

**Figure 3:**
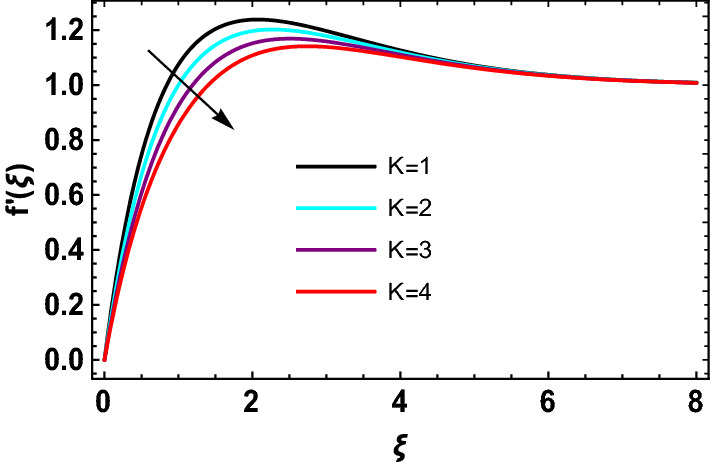
Impact of $K$ upon $f^{\prime} ( \xi )$.

**Figure 4:**
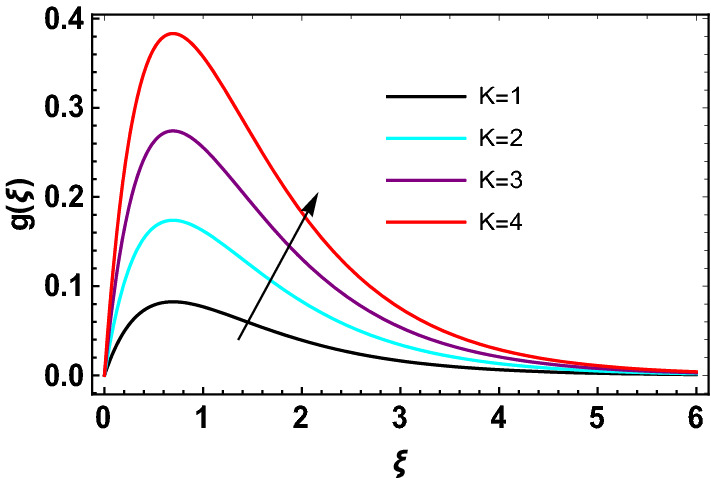
Impact of $K$ upon $g ( \xi )$.

**Figure 5:**
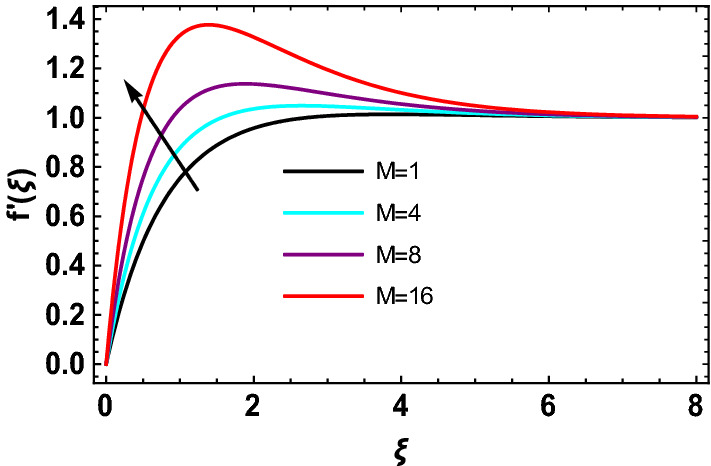
Impact of $M$ upon $f^{\prime} ( \xi )$.

**Figure 6:**
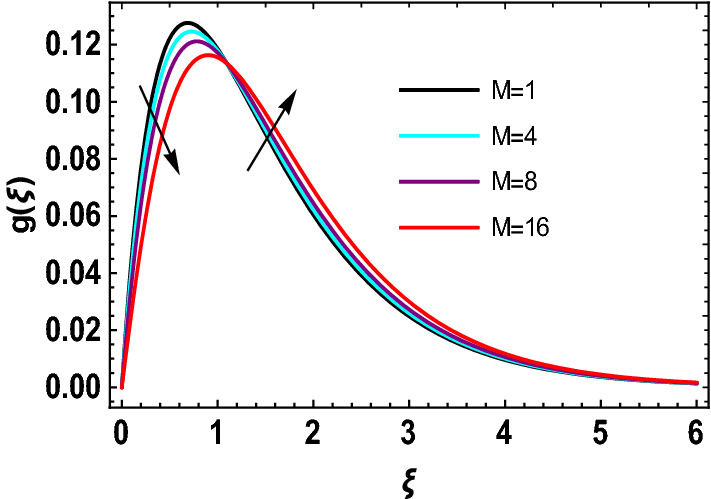
Impact of $M$ upon $g ( \xi )$.

**Figure 7:**
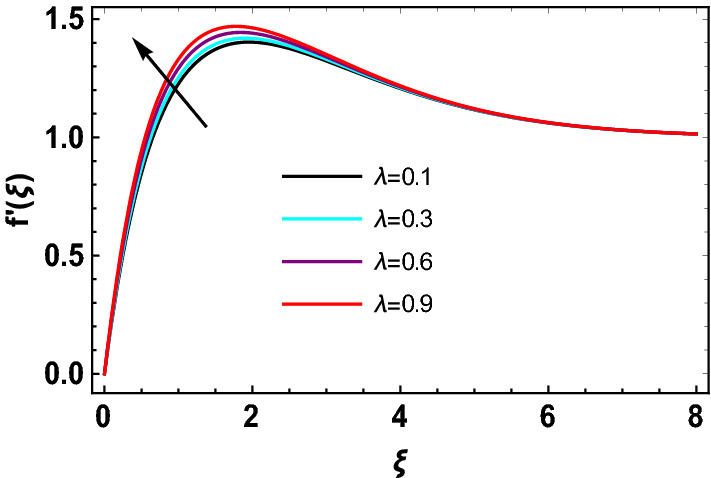
Impact of $\lambda$ upon $f^{\prime} ( \xi ).$

**Figure 8:**
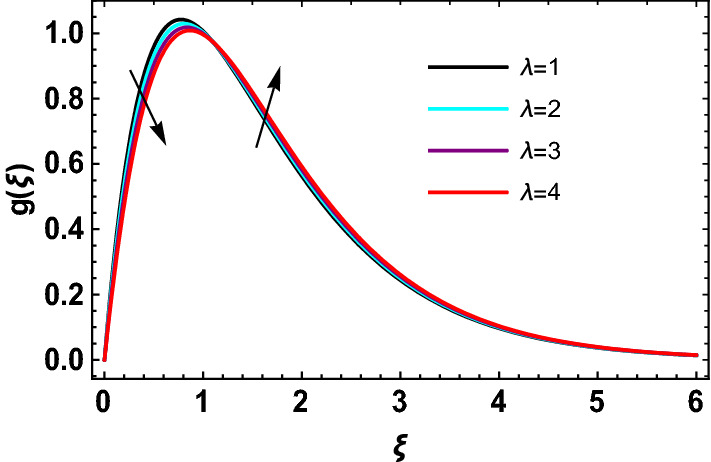
Impact of $\lambda$ upon $g ( \xi )$.

**Figure 9:**
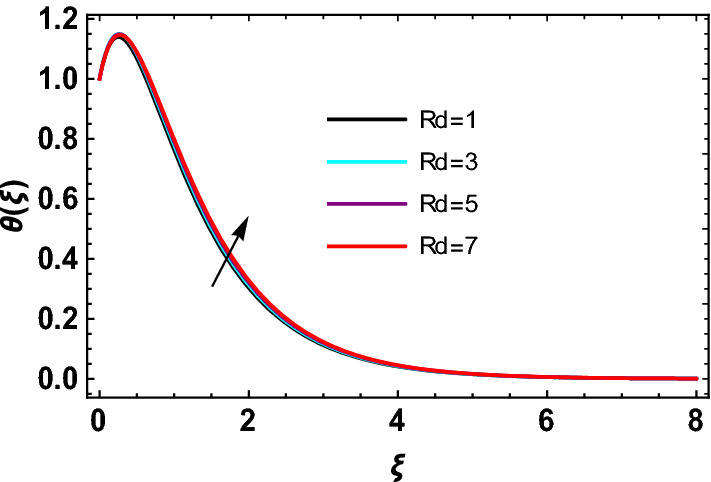
Impact of $Rd\,\,$ on $\theta  ( \xi )$.

**Figure 10:**
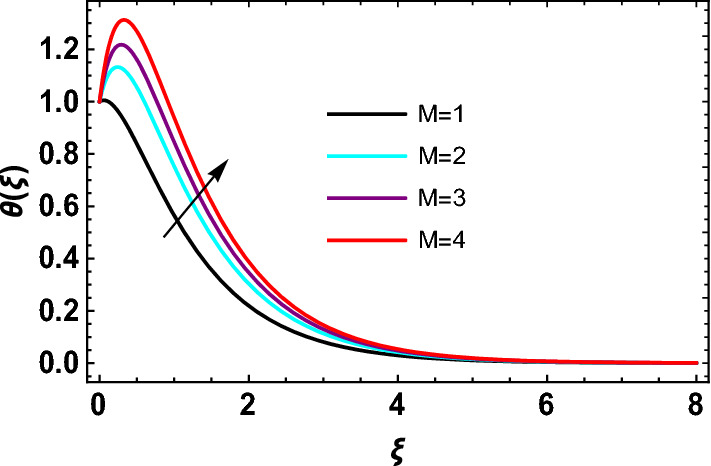
Impact of $M$ on $\theta  ( \xi )$.

**Figure 11:**
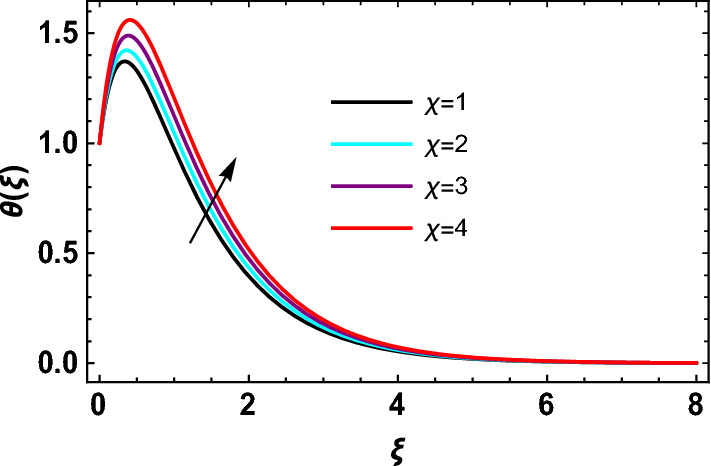
Impact of $\,\chi$ on $\theta  ( \xi )$.

**Figure 12:**
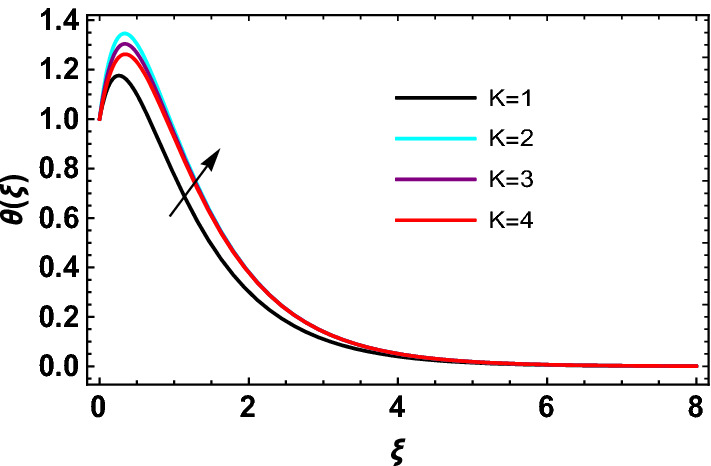
Impact of $K\,$ on $\theta  ( \xi )$.

**Figure 13:**
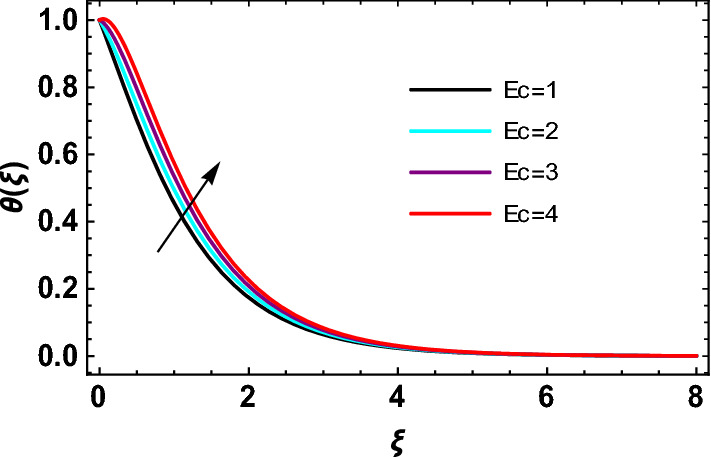
Impact of $Ec\,\,$ on $\theta  ( \xi )$.

**Figure 14:**
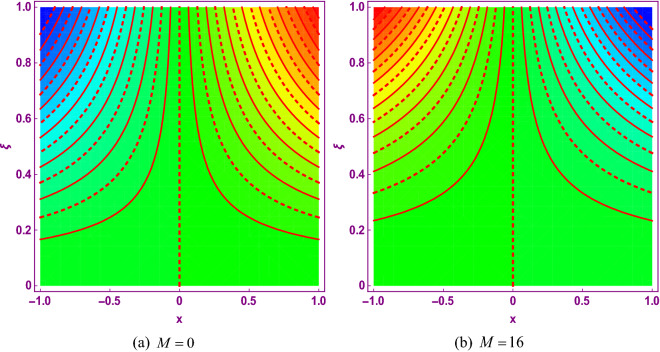
Streamlines patterns for $M = 0$ and $M = 16$.

This study explores the MHD natural convective micropolar hybrid nanofluid flow past a stretching plate. The fluid has influenced by thermal radiations and Joule heating effects. The plate has placed vertical in a permeable medium with suction and injection effects. The parameters that have influenced the current investigation have been discussed in following paragraphs. Figure 1 describes the fluid flow problem. Figure 2 presents the convergence analysis of the solution method for current investigation. It is to be noticed that throughout the discussion of results with the help of graphical view, we have used fixed computational values for substantial factors such as $\Pr = 6.2,\,\,Rd = 1,\,\,Q = Ec = K = 0.5,\,\,\lambda = 0.5,\,\,\phi_{1} = \phi_{2} = 0.04$. The influence of different substantial parameters upon velocity $f^{\prime} ( \xi )$ and micro-rotational velocity $g ( \xi )$ profiles has depicted in Figs. 3, 4, 5, 6, 7, 8. In Figs. 3 and 4 the influence of micropolar parameter $K$ upon $f^{\prime} ( \xi )$ and $g ( \xi )$ has presented. Since with growing values of $K$ extra resistive force is experienced by the fluid motion in the linear direction of motion while the rotational motion is supported in this process. Hence higher values of $K$ weaken the values of $f^{\prime} ( \xi )$ and upsurge the values of $g ( \xi )$ as depicted in Figs. 3 and 4 respectively. Figure 5 presents that augmenting values of magnetic factor $M$ results in the upsurge of momentum boundary layer strength. Hence growth in magnetic effects augments the fluid velocity of flow system. In case of micro-rotational velocity there is two-fold behavior of $M$ upon $g ( \xi )$. In the interval $0 < \xi < 1$ there is a reduction in the values of $g ( \xi )$ whereas in the domain $1 < \xi < 6$ the strength of momentum boundary layer augments that upsurges the micro-rotational velocity of fluid motion as illustrated in Fig. 6. For growth in mixed convection parameter $\lambda$ the buoyancy forces augment, that supports the linear velocity of fluid flow system. Hence upsurge in $\lambda$ results an augmentation in $f^{\prime} ( \xi )$ as depicted in Fig. 7. In the similar manner as for magnetic effects again there is a two-fold behavior of variations in $\lambda$ against the values of $g ( \xi )$. Clearly in the interval $0 < \xi < 1$ there is a reduction in the values of $g ( \xi )$, while in the domain $1 < \xi < 6$ the thickness of momentum boundary layer augments that upsurges the micro-rotational velocity of fluid motion as depicted in Fig. 8. Impact of different substantial factors upon thermal flow profiles has been depicted in Figs. 9, 10, 11, 12, 13. Variations in thermal profiles subject to changes in radiation factor $Rd$ are illustrated in Fig. 9. The numerical values of $Rd$ present the input comparison of thermal transmission via conduction of transfer via thermal radiation; hence with augmentation in $Rd$ maximum heat diffusion occurs. Therefore, with upsurge in the values of $Rd$ thermal profiles augment as described in Fig. 9. Application of the effects of magnetic factor $M$ to a fluid motion produces Lorentz force that strongly opposes the motion in reverse direction. Hence growth in $M$ generates more skin friction that results an upsurge in the thermal profiles as illustrated in Fig. 10. The variations in thermal flow with respect to heat generation and absorption factor $\chi$ are depicted in Fig. 11. It is obvious that for positive values of $\chi$ a heat generation “that is source” is found in the fluid flow. Hence growth in $\chi$ results an augmentation in thermal flow profiles as presented in Fig. 11. The influence of micro-polar factor $K$ upon thermal profiles has depicted in Fig. 12. Since with growth in $K$ the heating effects augment within the layer of thermal boundary region, hence higher values of $K$ upsurge the values of thermal profiles. Figure 13 depicts variations in thermal profiles in response of changes in Eckert number $Ec$. Actually, $Ec$ is employed to establish the effects of self-heating of fluid flow due to influence of dissipation. Hence, due to growth in $Ec$ the thermal characteristics of micropolar fluid upsurge. Figure 14 shows the streamlines pattern for different values of $M$. Here, it is noted that with the increasing $M$, the streamlines patterns also increase. In Table 1 the numerical calculations for thermophysical characteristics are illustrated. Table 2 shows the comparison $f^{\prime\prime} ( 0 )$ with published results. Here, a great agreement is found with those published results. In Table 3 the impact of various substantial factors over skin friction of fluid flow system has been portrayed in numerical manners. Since the fluid motion has upsurge with expansion in magnetic effects and mixed convection factor while it has declined with augmenting values of micropolar factor. Hence intensification in magnetic strength and mixed convection factors has declined the skin friction and has upsurge with higher values of micropolar parameter. Actually, with upsurge in magnetic strength and mixed convection, lesser fraction has experienced by the micropolar nanoparticles at the surface of stretching sheet that has declined skin friction in the closed locality of stretching plate. The numerical impacts of different substantial factors upon fluid’s thermal flow rate have been depicted in Table 4. Since for growth in magnetic effects, radiation factor and Eckert number and in strength of heat source the thermal flow of micropolar fluid augment, hence the Nusselt number has increased with intensification in the values of these parameters.

**Table 2:** Comparison of $f^{\prime\prime} ( 0 )$ with published results of Zaib et al.^61^, when $\lambda = 0$ and all other parameters are zero.

$\Pr$	Zaib et al.^61^	Present results
0.7	1.7063	1.7063
1.0	1.6754	1.6754
7.0	1.5179	1.5179
10.0	1.4928	1.4928

**Table 3:** Influences of $M$,$\lambda$ and $K$ on $C_{f}$.

$M$	$\lambda$	$K$	$C_{f}$
0.5			− 0.274370
1.0			− 0.345012
1.5			− 0.415654
2.0			− 0.486296
	0.1		− 0.246938
	0.2		− 0.253796
	0.3		− 0.260654
	0.4		− 0.267512
		0.1	0.216446
		0.2	0.256543
		0.3	0.290717
		0.4	0.318968

**Table 4:** Influences of $M$, $Rd$, $Ec$ and $\chi$ on $Nu$.

$M$	$Rd$	$Ec$	$\chi$	$Nu$
0.5				0.290790
1.0				0.350868
1.5				0.410947
2.0				0.471025
	0.1			0.290790
	0.3			0.295220
	0.5			0.299650
	0.7			0.304079
		1.0		0.539396
		2.0		1.036606
		3.0		1.533817
		4.0		2.031028
			1.1	0.284866
			1.2	0.290790
			1.3	0.290790
			1.4	0.302638

## Conclusion

This study explores mixed convective MHD micropolar water-based hybrid nanofluid flow containing silver and alumina past a flat surface. The hybrid nanofluid flow is taken under viscous dissipation, thermal radiation and Joule heating effects. The surface is placed vertical in a permeable medium with suction and injection effects. Specific similarity variables have used to transform the set of modeled equations to dimension-free form and then solved by HAM. Following main points have been concluded:

1. Fluid motion upsurges with the increase is magnetic parameter and mixed convection parameter while reduces with the growth in micropolar factor.
2. Micro-rotational velocity of fluid upsurges with higher values of micropolar factor.
3. For growth in mixed convection and magnetic factors the micro-rotational motion exhibits two-fold behavior. In the interval $0 < \xi < 1$ there is a reduction while in the domain $1 < \xi < 6$ there is a growth in micro-rotational motion for higher values of both parameters.
4. Thermal flow behavior is augmenting for expended values of magnetic effects, radiation factor, Eckert number and heat source strength.
5. The intensification in magnetic strength and mixed convection factors has declined the skin friction and has upsurge with higher values of micropolar parameter.
6. The Nusselt number has increased with the intensification in magnetic effects, radiation factor and Eckert number.

## Data Availability

The data related to this manuscript are within the manuscript.
